# The causal relationship between 41 inflammatory cytokines and hypothyroidism: bidirectional two-sample Mendelian randomization study

**DOI:** 10.3389/fendo.2023.1332383

**Published:** 2024-01-22

**Authors:** Rui Lai, Bingzun Yin, Ziyang Feng, Xinmin Deng, Xiaofeng Lv, Yumei Zhong, Dezhong Peng

**Affiliations:** ^1^ School of Acupuncture and Tuina, Chengdu University of Traditional Chinese Medicine, Chengdu, China; ^2^ The Fourth Clinical Medical College, Guangzhou University of Chinese Medicine, Shenzhen, China; ^3^ School of Clinical Medicine, Chengdu University of Traditional Chinese Medicine, Chengdu, China; ^4^ Chengdu Integrated TCM & Western Medicine Hospital/Chengdu First People’s Hospital, Chengdu, China

**Keywords:** Mendelian randomization, inflammatory, cytokines, hypothyroidism, causal relationship

## Abstract

**Objective:**

Investigating the association between inflammatory cytokines and hypothyroidism remains challenging due to limitations in traditional observational studies. In this study, we employed Mendelian randomization (MR) to assess the causal relationship between 41 inflammatory cytokines and hypothyroidism.

**Method:**

Inflammatory cytokines in 30,155 individuals of European ancestry with hypothyroidism and in a GWAS summary containing 8,293 healthy participants were included in the study for bidirectional two-sample MR analysis. We utilized inverse variance weighting (IVW), weighted median (WM), and Mendelian randomization-Egger (MR-Egger) methods. Multiple sensitivity analyses, including MR-Egger intercept test, leave-one-out analysis, funnel plot, scatterplot, and MR-PRESSO, were applied to evaluate assumptions.

**Results:**

We found evidence of a causal effect of IL-7 and macrophage inflammatory protein-1β (MIP-1β) on the risk of hypothyroidism, and a causal effect of hypothyroidism on several cytokines, including granulocyte colony-stimulating factor (G-CSF), IL-13, IL-16, IL-2rα, IL-6, IL-7, IL-9, interferon-γ-inducible protein 10 (IP10), monokine induced by interferon (IFN)-γ (MIG), macrophage inflammatory protein-1β (MIP-1β), stem cell growth factors-β (SCGF-β), stromal cell derived factor-1α (SDF-1α), and tumor necrosis factor-α (TNF-α).

**Conclusion:**

Our study suggests that IL-7 and MIP-1β may play a role in the pathogenesis of hypothyroidism, and that hypothyroidism may induce a systemic inflammatory response involving multiple cytokines. These findings may have implications for the prevention and treatment of hypothyroidism and its complications. However, further experimental studies are needed to validate the causal relationships and the potential of these cytokines as drug targets.

## 1 Introduction

Hypothyroidism is a common endocrine disorder characterized by high levels of thyroid-stimulating hormone (TSH) and low levels of thyroid hormones, such as triiodothyronine (T3), thyroxine (T4), free triiodothyronine (FT3) and free thyroxine (FT4). The prevalence of hypothyroidism ranges from 0.2% to 5.3% (1, 2). Thyroid dysfunction affects various systems, such as metabolic, cardiovascular, neurological, and immune systems, and increases the risk of mortality (3).

In recent years, evidence has accumulated that inflammation is involved in the progression of hypothyroidism (4, 5). C-reactive protein (CRP), a hepatically synthesized acute-phase reactant, has a response to inflammatory cytokines, including IL-6 (6). Numerous studies have identified a notable positive association between TSH and CRP (7–9). Some cytokines, such as tumor necrosis factor-α (TNF-α) and IL-6, may inhibit the hypothalamic-pituitary-thyroid axis (10, 11). Additionally, it is worth noting that IL-6 levels have the potential to serve as an indicator of the extent of hypothyroidism. This is due to the observed negative correlation between serum IL-6 levels and FT4 levels in individuals diagnosed with autoimmune hypothyroidism (12). The administration of Levothyroxine (LRT) has the potential to decrease the levels of pro-inflammatory cytokines, specifically IL-6 and TNF-α (13). These findings suggest a possible bidirectional relationship between increased inflammatory markers and altered thyroid function; however, the effect of hypothyroidism treatment on systemic inflammation is inconsistent. For example, a study by Díez et al (14)., found no statistically significant decrease in elevated levels of TNF-α and TNF-α receptors following the restoration of thyroid function in individuals with hypothyroidism.

Therefore, the potential causal relationship between inflammatory cytokines and hypothyroidism requires further investigation. Most of the evidence on the relationship between inflammatory cytokines and hypothyroidism comes from observational studies, which are prone to reverse causality, selection bias, and confounding factors. Therefore, more research using innovative methods is needed.

Mendelian randomization (MR) is one such technique that utilizes genetic variation as an instrumental variable to assess causal relationships between exposures and specific outcomes (15).

## 2 Method

### 2.1 Data sources

The two datasets included in this MR research were obtained from publicly accessible aggregated genome-wide association study (GWAS) data. The hypothyroidism data were obtained from the MRCIEU GWAS database (https://gwas.mrcieu.ac.uk/), which includes 30,155 cases and 379,986 European pedigree controls. For data on inflammatory factors, we obtained genetic predictors from the Young Finns Cardiovascular Risk (YFS) study, and the Finnish Cardiovascular Risk Study 1997 and 2002 (FINRISK) (16), which included up to 8,293 Finnish participants.

Since all the data were derived from publicly accessible studies, our research did not require patient consent or ethical clearance.

### 2.2 Study design

Our study design was divided into two main steps: first, we performed a two-sample analysis with 41 inflammatory factors as exposure and hypothyroidism as outcome, respectively. Then, a two-sample analysis was performed with hypothyroidism as the exposure and 41 inflammatory factors as the outcome, respectively.

### 2.3 Instrumental variable selection

**Figure 1:**
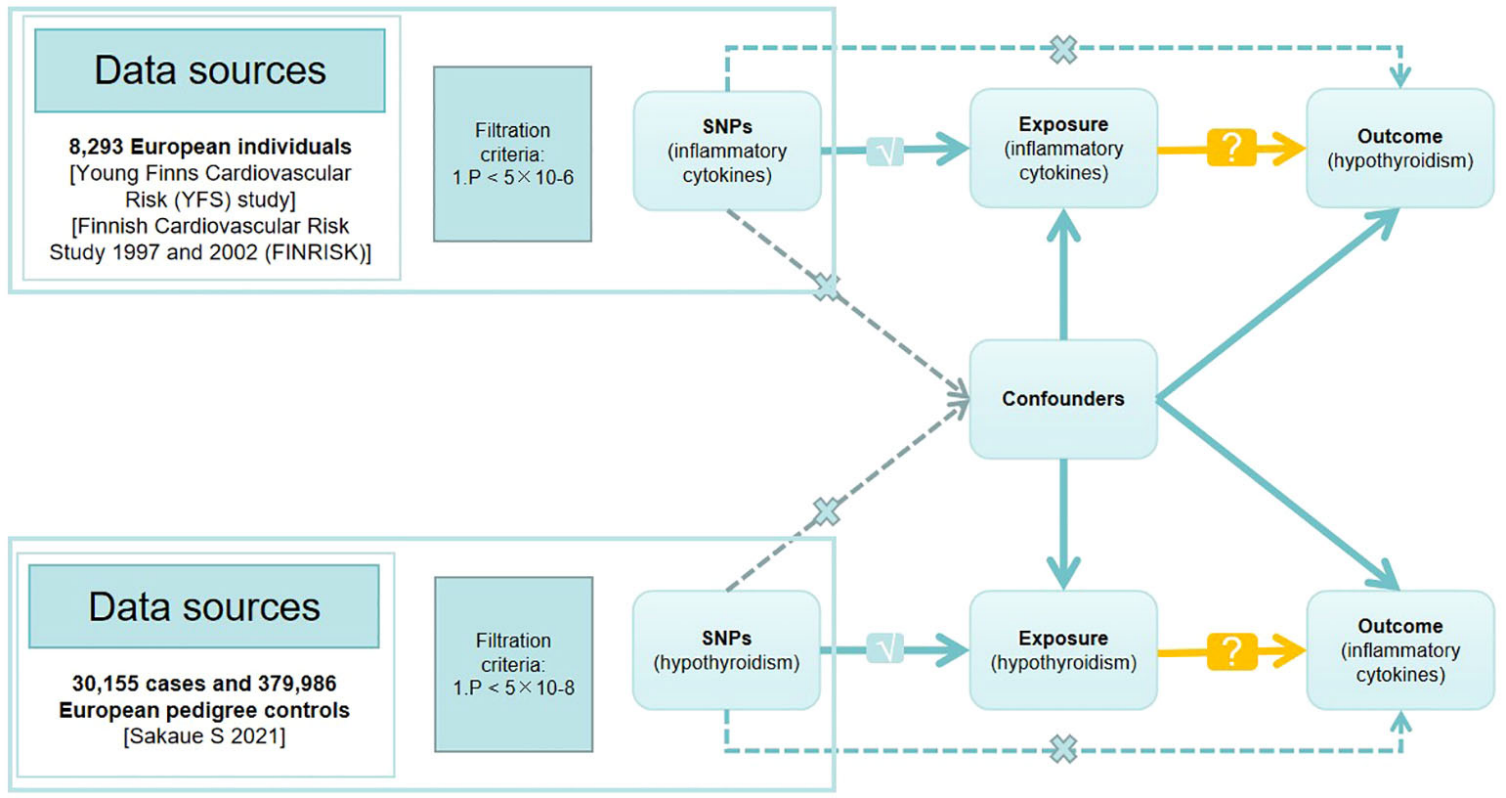
Directed acyclic graph of the Mendelian randomization (MR) framework investigating the causal relationship between exposure and outcome. The ‘×’ means that genetic variants are not associated with confounders or cannot be directly involved in outcome but via the exposure pathway. The ‘√’ means that genetic variants are highly correlated with exposure. SNPs, single-nucleotide polymorphisms.

In MR studies, genetic variants frequently serve as instrumental variables (IVs). Three critical assumptions must be met to obtain reliable casual estimates in MR studies: 1) IVs must show a high correlation with the exposure; 2) IVs must not be connected to any possible confounders that could skew the results of the exposure-outcome link; 3) IVs should only affect the result by means of exposure (17, 18). **Figure 1** depicts a detailed description.

The genome-wide significance criterion of p<5 × 10-8 was employed to identify SNPs that were significantly linked to both hypothyroidism and inflammatory cytokines. Since only a few SNPs were found, we adopted a higher cutoff (p<5 × 10-6) for inflammatory cytokines as the exposure. After that, we used SNP clustering to eliminate linkage disequilibrium (LD) with a 10,000 kb window size and an R2<0.001 criterion. Subsequently, we checked all the exposure (hypothyroidism) related SNPs in the PhenoScanner database (http://www.phenoscanner.medschl.cam.ac.uk/) to identify any SNPs associated with potential confounders (BMI, smoking, alcohol consumption) and the outcome. We extracted SNP effects from the outcome GWAS dataset and harmonized the impact of the exposure and outcome. We excluded palindromic SNPs with ambiguous results (EAF >0.42). To find and eliminate any outliers, we used the MR-Pleiotropy Residual Sum and Outlier (MR-PRESSO) technique (19). Finally, we assessed instrumental strength using the F-statistic ( $F=\frac{N-k-1}{k}\times \frac{R^{2}}{1-R^{2}}$ ), following the procedures outlined by Burgess and Thompson (20).

## 3 Mendelian randomization analyses

For our MR analyses, we employed R (version 4.2.2) packages, namely “TwoSampleMR” (version 0.5.6) and “MR-PRESSO” (version 1.0).

We assessed the association between inflammatory factors and hypothyroidism using three methods: inverse variance weighted (IVW), weighted median (WM), and Mendelian randomization-Egger (MR-Egger) methods. IVW method offers reliable and consistent results under the assumption that all genetic variants used as instruments are legitimate instrumental variables valid IVs (21). Conversely, MR-Egger regression yields reliable estimates, particularly in cases where all the genetic variants under consideration are invalid IVs. The weighted median approach generates consistent appraisals, requiring at least half of the weights to be derived from accurate IVs (22). Our primary result was based on the IVW method, while MR-Egger and WM approaches were used to assess the reliability and stability of the results.

We used the MR-Egger intercept test to detect horizontal pleiotropy, with a P-intercept >0.05 indicating the absence of such pleiotropy. We further employed the IVW method and Egger regression to evaluate heterogeneity, with P<0.05 indicating its presence. Cochran’s Q statistic was used to assess heterogeneity (23). Moreover, we conducted a leave-one-out analysis to investigate whether a single SNP was driving the causal association.

### 3.1 The effect of inflammatory cytokines on hypothyroidism

When the cutoff value for genome-wide significance was established at 5 × 10-8, only 9 of the 41 available inflammatory cytokines had 3 or more SNPs. Therefore, a higher criterion (P< 5 × 10-6) was employed to guarantee an adequate number of SNPs for further MR analysis. The F-statistic values of the SNPs associated with each inflammatory cytokine were higher than 10, indicating less weak instrumental bias (**Supplementary Table 1**).

**Figure 2:**
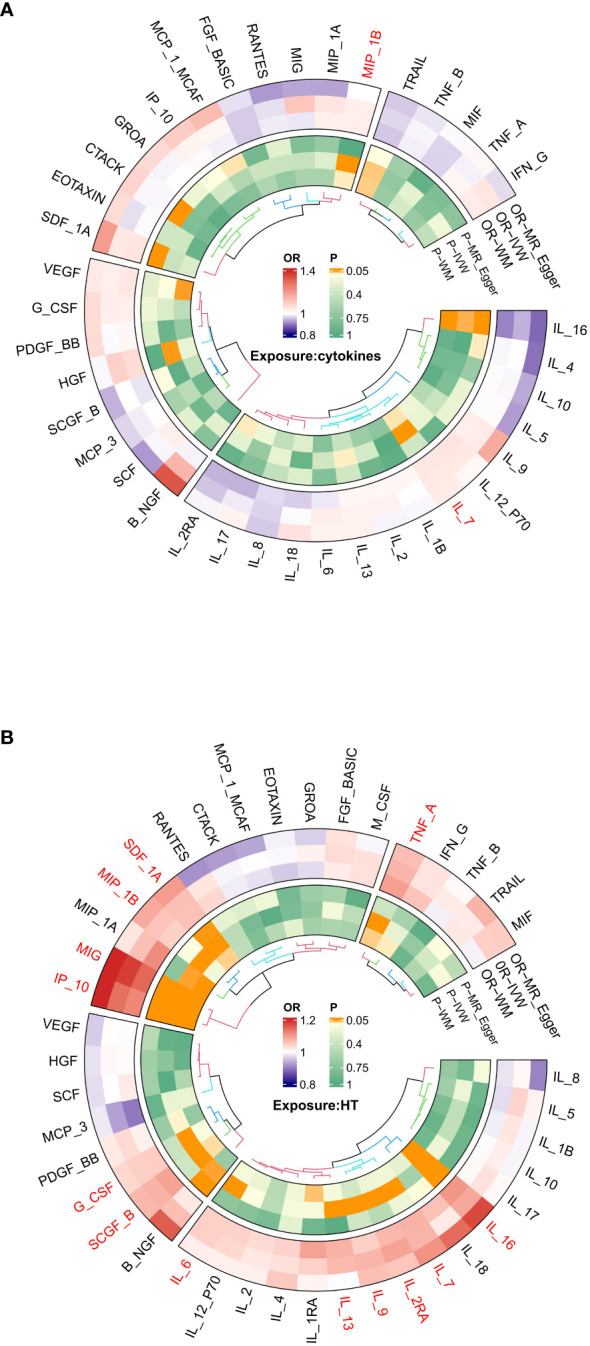
**(A)** Ring heat map of Mendelian randomization analysis of the causal effect of inflammatory cytokines on hypothyroidism. **(B)** Ring heat map of Mendelian randomization analysis of the causal effect of hypothyroidism on inflammatory cytokines.

**Figure 3:**
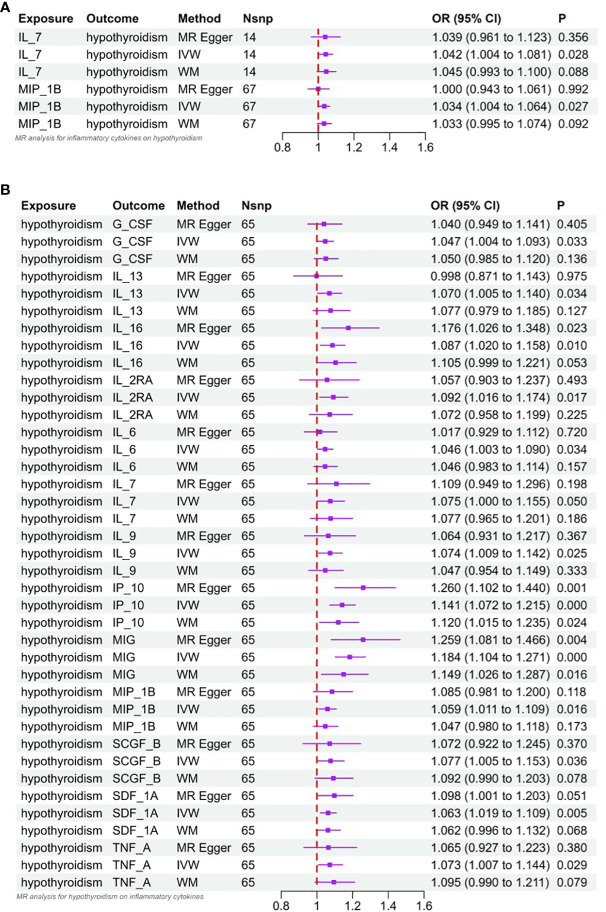
**(A)** Forest plot of Mendelian randomized analysis of causal effects of inflammatory cytokines on hypothyroidism. **(B)** Forest plot of Mendelian randomized analysis of causal effects of hypothyroidism on inflammatory cytokines.

Interleukin-l receptor antagonist (IL-1rα) and M-CSF still failed to be extracted as valid instrumental variables. In **Figures 2A** and **3A**, the primary MR analyses of inflammatory cytokines are displayed. By the IVW method, it was found that the level of IL-7 was associated with increased odds of developing hypothyroidism (IVW-OR: 1.042, 95% CI: 1.004-1.081, p = 0.028). Though the WM technique and MR Egger analysis were unable to identify a statistically significant link, they revealed a similar tendency (WM-OR: 1.045, 95% CI: 0.993-1.100, p = 0.088; MR Egger-OR: 1.039, 95% CI: 0.961-1.123, p = 0.356). The same causal relationship was found between MIP-1β and hypothyroidism (IVW-OR: 1.034, 95% CI: 1.004-1.064, p = 0.027). Similarly, the WM technique and MR Egger analysis were unable to identify a statistically significant link, they revealed a similar tendency (WM-OR: 1.033, 95% CI: 0.995-1.074, p = 0.092; MR Egger-OR: 1.000, 95% CI: 0.943-1.061, p = 0.992). **Supplementary Figures 1**–**3** illustrate the scatter plot, funnel plots, and leave-one-out analyses of IL-7 and MIC-1β in hypothyroidism.

According to Cochrane’s Q (Q_pval > 0.05), there was no evidence of heterogeneity in the correlation between IL-7 and MIP-1β, and no outlier SNPs were found using the MR-PRESSO technique. In addition, the MR-Egger intercept did not find evidence of horizontal pleiotropy (P = 0.935; P = 0.218) (**Supplementary Table 2**). Scatterplots, funnel plots, and leave-one-out analyses of Mendelian randomization analyses of the role of IL-7 and MIP-1β in hypothyroidism are shown in **Supplementary Figures 4**–**6**.

### 3.2 Effect of hypothyroidism on inflammatory cytokines

When TNF-β was used as an outcome, we extracted 61 SNPs as IVs for hypothyroidism. whereas additionally when 40 inflammatory cytokines were used as outcomes, we extracted 65 significant SNPs as IVs for hypothyroidism.

The results of MR analysis regarding the causal relationship between hypothyroidism and inflammatory cytokines are shown in **Figures 2B** and **3B**. The results of the IVW method showed a suggestive correlation between hypothyroidism and elevated levels of colony-stimulating factor (G-CSF), IL-13, IL-16, IL-2rα, IL-6, IL-7, IL-9, IP10, interferon-γ-inducible protein 10 (IP10), monokine induced by interferon (IFN)-γ (MIG), macrophage inflammatory protein-1β (MIP-1β), stem cell growth factors-β (SCGF-β), stromal cell derived factor-1α (SDF-1α), and TNF-α.

In the sensitivity analysis, Cochran’s Q test (Q_pval > 0.05) showed that there was no heterogeneity in the validated IVs used to estimate the effect of hypothyroidism on inflammatory cytokines. In addition, there was no evidence of horizontal pleiotropy (MR-Egger interception P > 0.05) in any of the MR analyses of the effect of hypothyroidism on inflammatory cytokines (**Supplementary Table 2**). Scatter plots, funnel plots, and leave-one-out analyses of inflammatory cytokines suggestive of relevance are shown in **Supplementary Figures 2** and **3**.

## 4 Discussion

This study employed a bidirectional two-sample MR approach to investigate the potential causal association between inflammatory cytokines and hypothyroidism. Our results suggested that IL-7 and MIP-1β might cause hypothyroidism. Conversely, when we treated hypothyroidism as an exposure factor, we detected a causal association between hypothyroidism and 13 inflammatory cytokines (IL-13, IL-16, IL-2rα, IL-6, IL-7, IL-9, G-CSF, SCGF-β, IP-10, MIG, MIP-1β, SDF-1α, and TNF-α).

Several studies have shown that patients with hypothyroidism have increased levels of inflammatory cytokines (14, 24–30). Chronic autoimmune thyroiditis (CAT) is the main cause of hypothyroidism in iodine-sufficient countries (31), and cytokines are involved in autoimmune thyroid disease through various mechanisms. They are produced by inflammatory cells such as lymphocytes and by thyroid follicular cells (TFC) in the thyroid gland. In autoimmune hypothyroidism tissue samples, gene expression of IL-1, IL-2, IL-4, IL-6, IL-8, IL-10, IL-12, IL-13, IL-15, IL-16, IL-18, IFN-γ, TNFα, and some chemokines has been observed (32–35). Gene expression of IL-2, IL-4, IL-6, IL-8, IL-10, IL-13, IL-15, IL-16, IL-17, IFN-γ, and TNFα has also been detected in autoimmune thyroid disease-derived lymphocytes (infiltrating thyroid lymphocyte, ITL). Moreover, TFC can produce cytokines themselves. TFC can express mRNAs for IL-1, IL-6, IL-8, IL-12, IL-13, IL-15, IL-16, IL-17, IL-18, TNFα, MIP-1α, MIP-1β, MCP-1, RANTES, IP-10, and MIG (32, 34–37).

The clinical status of the patient may influence the type of immune response, with Th1-type T cells predominating in hypothyroid patients, whereas euthyroid patients with autoimmune thyroiditis have a mixed Th1/Th2 response (38, 39). The accumulation of Th1 lymphocytes stimulates the overproduction of IFN-γ and TNF-α, which further activate various cells to secrete IFN-γ, creating a positive feedback loop at the site of inflammation and resulting in elevated levels of IFN-γ (39, 40). IP-10 is a chemokine-induced by IFN-γ stimulation. It can be secreted by IFN-γ-activated thyroid cells. Therefore, high levels of IP-10 in peripheral blood indicate a Th1-polarized immune response. Previous studies have shown that patients with autoimmune thyroiditis have increased circulating IP-10 levels (41).

Tayde et al. (41) reported that hypothyroid patients had higher levels of TNF-α, IL-6, and CRP than healthy controls and that levothyroxine treatment reduced but did not normalize these cytokines. Another study also found that levothyroxine treatment modulated the inflammatory profile of hypothyroid patients, decreasing pro-inflammatory cytokines and increasing anti-inflammatory cytokines (13). Antunes et al. (42) showed that TSH activated IL-6, a pro-inflammatory cytokine, in preadipocytes via the cAMP protein kinase A signaling pathway. Whetsell et al. (43) demonstrated that TSH induced TNF-α in myeloid cells. Moreover, several *in vitro* studies have indicated that TSH can affect cytokine release in different cell types (42–46). Therefore, lower TSH levels may lead to lower levels of several inflammatory factors. Conversely, recombinant human TSH administration can cause a significant increase in blood cytokine levels in patients with differentiated thyroid cancer (47). IFN-γ can inhibit the transcription of the TSH-R gene, which may result in a general suppression of thyroid function *in vivo* (48).

Thyroid dysfunction is associated with IFNα treatment for malignant tumors or chronic hepatitis (49). A long-term study on the effects of IFNα treatment on thyroid function showed that high levels of thyroid autoantibodies, especially the combination of TPO and TG antibodies, after IFNα treatment were predictive factors for thyroid dysfunction (50). IFNβ treatment for multiple sclerosis may also cause thyroid dysfunction, but this is controversial (51, 52). However, IFN-γ application in humans does not affect thyroid function or thyroid autoantibody production (53). The mechanism of cytokine therapy-induced thyroid dysfunction is unclear. One possibility is that some cytokines have broad immunomodulatory effects that trigger the development of autoimmune thyroid disease (54). Moreover, as mentioned earlier, cytokines can also directly interfere with TFC function and immune responses, which may be a major factor in cytokine therapy-related thyroid dysfunction. Steroid therapy can increase IL-4 and IL-10 levels, suggesting that these anti-inflammatory cytokines may contribute to disease remission (55).

We conducted the first MR study to assess the causal relationship between hypothyroidism and 41 inflammatory cytokines. However, our study had some limitations. First, we used an MR design that excluded known confounders, but unmeasured confounders may still bias the results, which may influence both hypothyroidism and cytokine levels. Second, we focused only on individuals of European ancestry, which may reduce the applicability of our findings to other populations. Therefore, our results should be interpreted with caution for broader populations. In addition, because of the large geographic variation in the incidence of hypothyroidism itself, it is subject to large geographic influences, even for individuals of the same European ancestry. Third, due to limitations in the raw GWAS data, we were unable to stratify the studies by sex. This is a major drawback, as hypothyroidism is more prevalent and severe in women than in men, and sex hormones may modulate the immune response and cytokine production. Therefore, our results may not capture the potential sex-specific effects of hypothyroidism on cytokine levels. Future studies should explore the possible interactions between sex, hypothyroidism, and cytokines, and adjust for sex as a potential confounder or effect modifier. Finally, hypothyroidism has a complex etiology with multiple subtypes, such as autoimmune, iodine deficiency, or drug-induced hypothyroidism, which were not considered in our MR analysis. These subtypes may have different mechanisms and consequences for the inflammatory response and cytokine levels. Therefore, our results may not reflect the heterogeneity and specificity of the relationship between hypothyroidism and cytokines. Future studies should classify hypothyroidism according to its etiology and subtype, and compare the effects of different subtypes on cytokine levels.

## 5 Conclusion

Our study investigated the potential causal relationship between 41 inflammatory cytokines and hypothyroidism using a bidirectional two-sample MR analysis technique. We report several strong associations, but further experimental validation is needed to assess the potential of these cytokines to be used as drugs to prevent hypothyroidism. Furthermore, the study of causality is advantageous for disease prevention, as well as for promoting the refinement and advancement of treatment. It is necessary to conduct larger epidemiological or metagenomic studies to attain a more comprehensive understanding of the observed correlation between the susceptibility of hypothyroidism in patients with specific inflammatory cytokines.

## Data availability statement

The original contributions presented in the study are included in the article/**Supplementary Material**. Further inquiries can be directed to the corresponding authors.

## Ethics statement

Ethical review and approval were not required for the study on human participants in accordance with the local legislation and institutional requirements. Written informed consent from the participant’s legal guardian/next of kin was not required to participate in this study in accordance with the national legislation and institutional requirements.

## Author contributions

RL: Conceptualization, Data curation, Formal analysis, Methodology, Writing – original draft, Writing – review & editing. BY: Visualization, Writing – original draft, Writing – review & editing. ZF: Data curation, Visualization, Writing – original draft. XD: Data curation, Visualization, Writing – original draft. XL: Data curation, Visualization, Writing – original draft. YZ: Supervision, Writing – review & editing. DP: Supervision, Writing – review & editing.
