# Supplementary material for: The causal relationship between 41 inflammatory cytokines and hypothyroidism: bidirectional two-sample Mendelian randomization study
Source: Front Endocrinol (Lausanne). 2024 Jan 22;14:1332383. doi: 10.3389/fendo.2023.1332383 (PMC10840409; doi:10.3389/fendo.2023.1332383)
Supplement: Supplementary file 1 [file DataSheet_1.docx]

**Supplementary material**

**Supplementary Figure 1. Scatter plots**
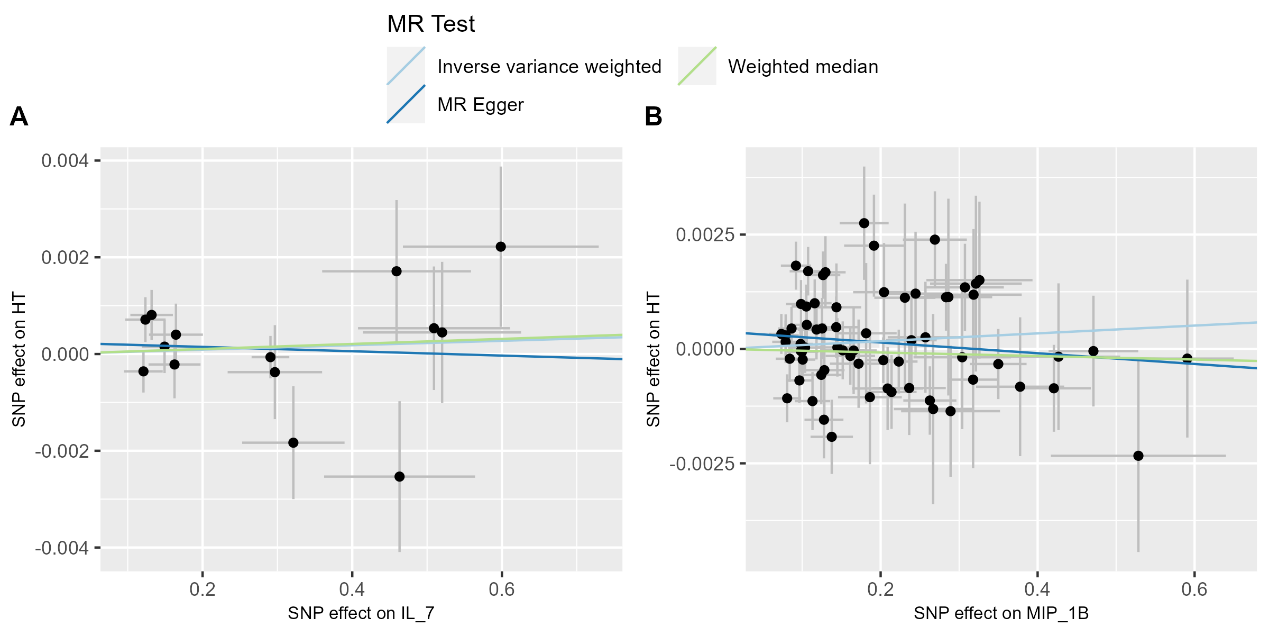
 **of inflammatory cytokines on hypothyroidism.**

The estimate of intercept can be interpreted as an estimate of the average pleiotropy of all single-nucleotide polymorphisms (SNPs), and the slope coefficient provides an estimate of the bias of the causal effect. (A) IL-7 on hypothyroidism. (B) MIP_1β on hypothyroidism.

**Supplementary Figure 2. Funnel plots of inflammatory cytokines on hypothyroidism.**


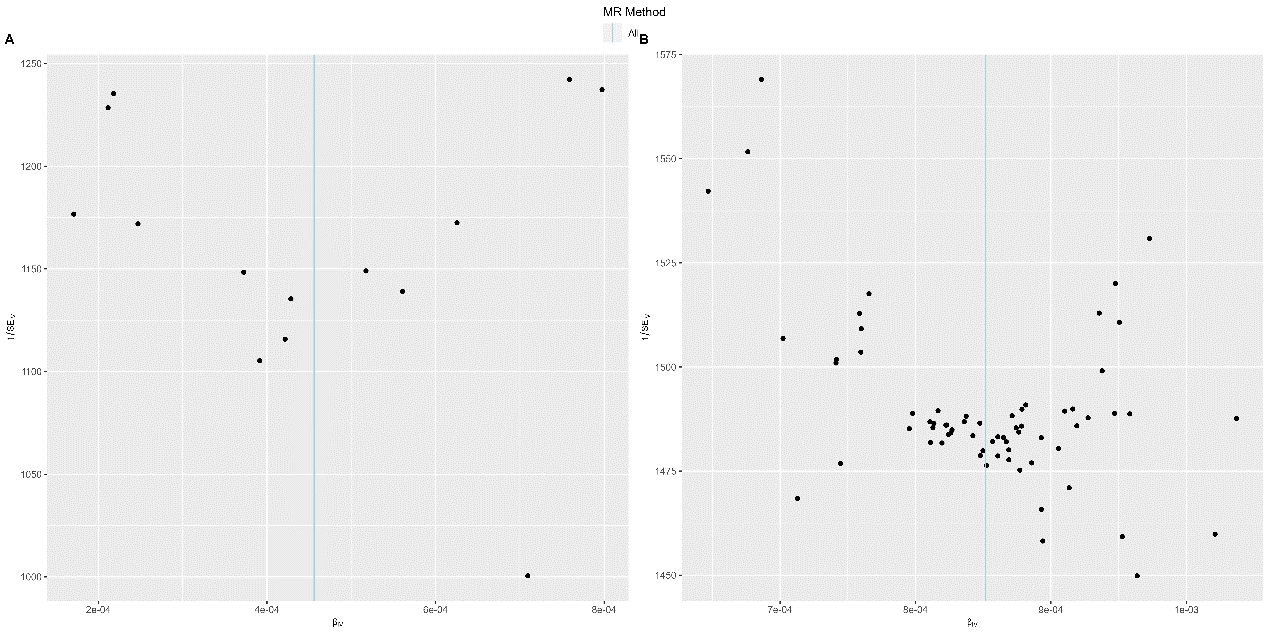


The x-axis represents β, and the y-axis represents 1/SE (standard error). (A) IL-7 on hypothyroidism. (B) MIP_1β on hypothyroidism.

**Supplementary Figure 3. Leave-one-out sensitivity analysis of inflammatory cytokines on hypothyroidism.**


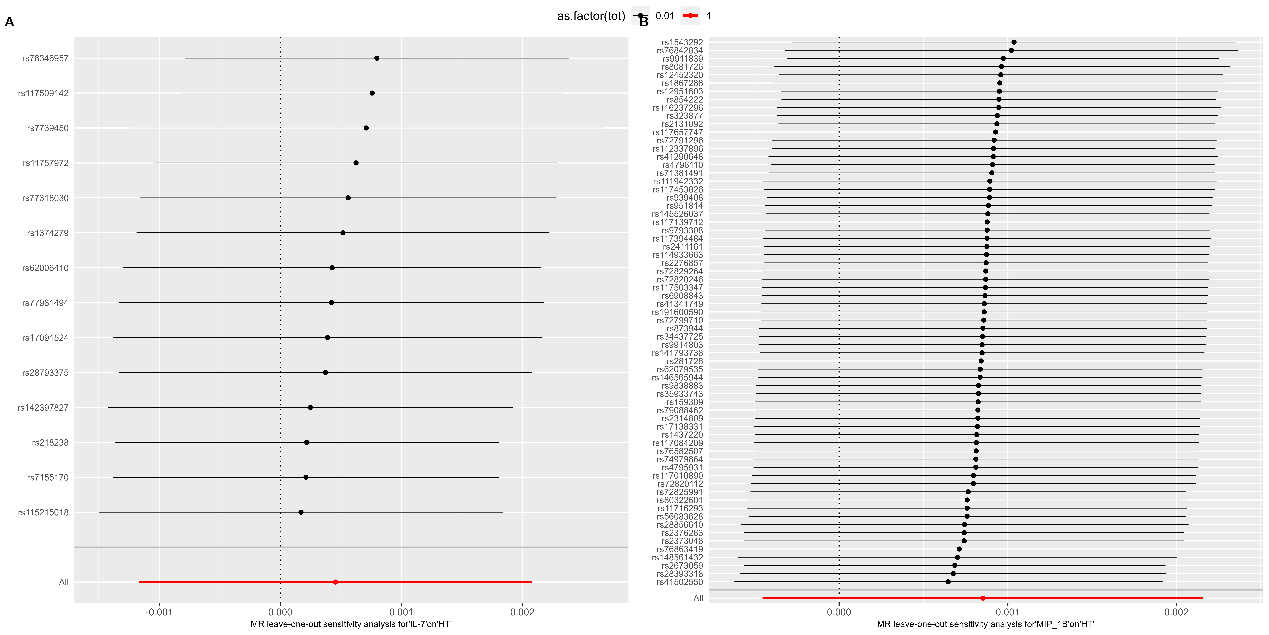


(A) IL-7 on hypothyroidism. (B) MIP_1β on hypothyroidism.

**Supplementary Figure 4. Scatter plots of hypothyroidism on inflammatory cytokines.**


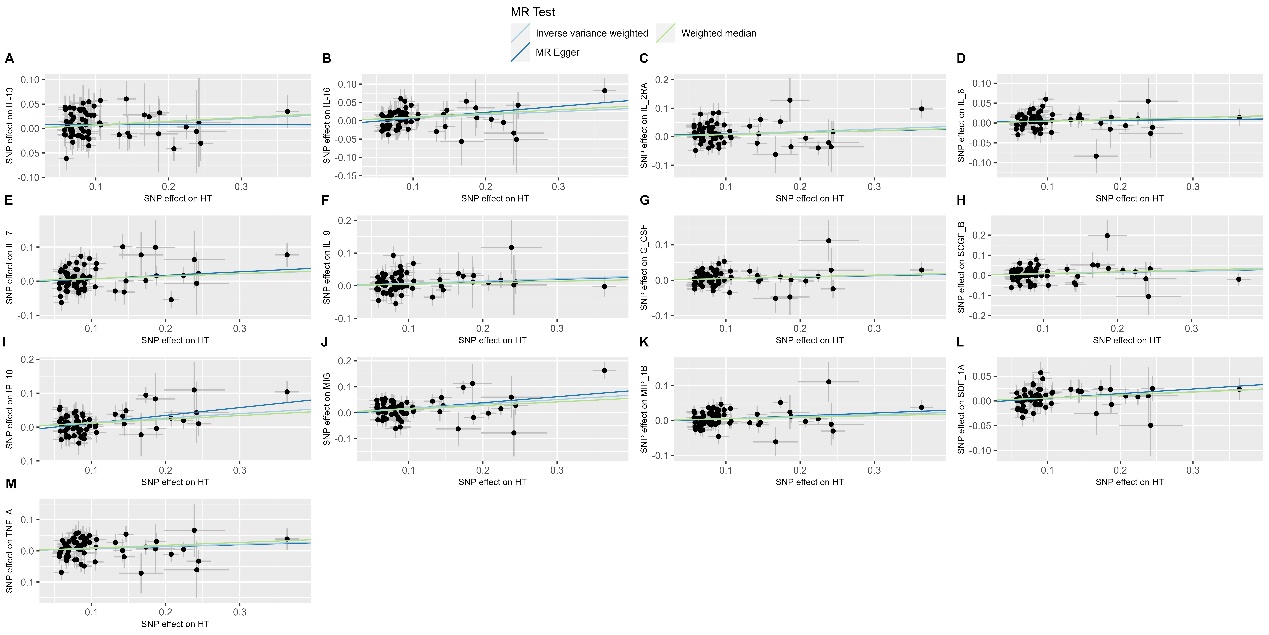


(A) Hypothyroidism on IL-13. (B) Hypothyroidism on IL-16. (C) Hypothyroidism on IL-2rα. (D) Hypothyroidism on IL-6. (E) Hypothyroidism on IL-7. (F) Hypothyroidism on IL-9. (G) Hypothyroidism on G-CSF. (H) Hypothyroidism on SCGF-β. (I) Hypothyroidism on IP-10. (J) Hypothyroidism on MIG. (K) Hypothyroidism on MIP-1β. (L) Hypothyroidism on MIP-1α. (M) Hypothyroidism on TNF-α.

**Supplementary Figure 5. Funnel plots of hypothyroidism on inflammatory cytokines.**


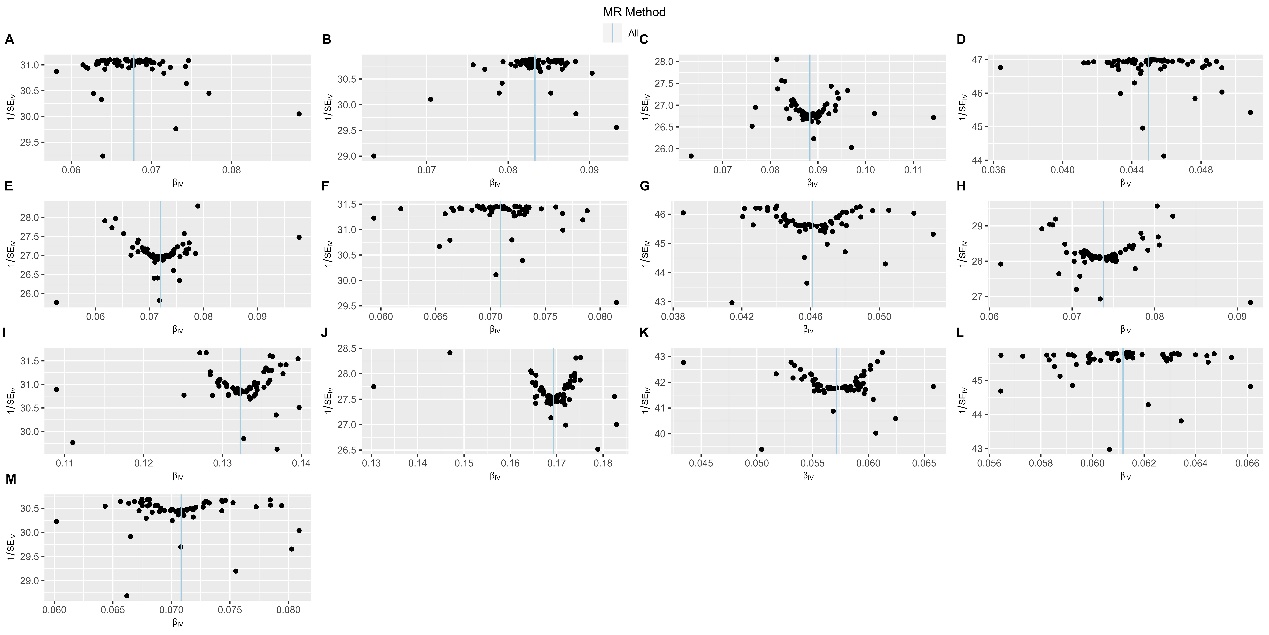


(A) Hypothyroidism on IL-13. (B) Hypothyroidism on IL-16. (C) Hypothyroidism on IL-2rα. (D) Hypothyroidism on IL-6. (E) Hypothyroidism on IL-7. (F) Hypothyroidism on IL-9. (G) Hypothyroidism on G-CSF. (H) Hypothyroidism on SCGF-β. (I) Hypothyroidism on IP-10. (J) Hypothyroidism on MIG. (K) Hypothyroidism on MIP-1β. (L) Hypothyroidism on MIP-1α. (M) Hypothyroidism on TNF-α.

**Supplementary Figure 6. Leave-one-out sensitivity analysis of hypothyroidism on inflammatory cytokines.**


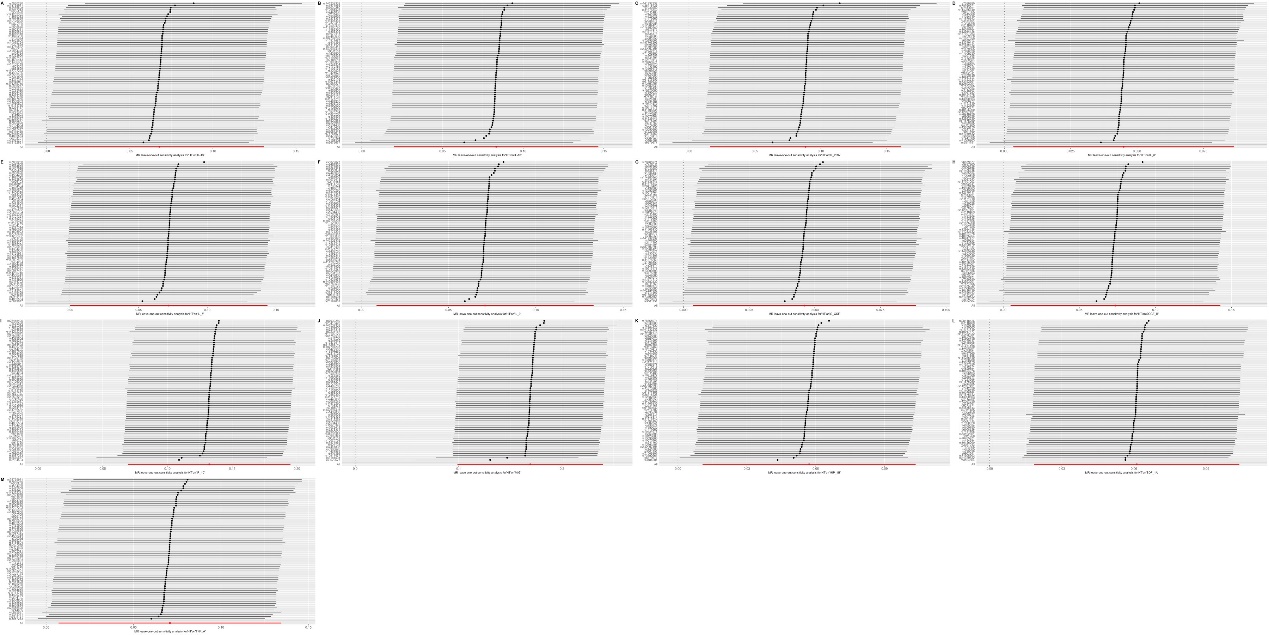


(A) Hypothyroidism on IL-13. (B) Hypothyroidism on IL-16. (C) Hypothyroidism on IL-2rα. (D) Hypothyroidism on IL-6. (E) Hypothyroidism on IL-7. (F) Hypothyroidism on IL-9. (G) Hypothyroidism on G-CSF. (H) Hypothyroidism on SCGF-β. (I) Hypothyroidism on IP-10. (J) Hypothyroidism on MIG. (K) Hypothyroidism on MIP-1β. (L) Hypothyroidism on MIP-1α. (M) Hypothyroidism on TNF-α.
