# Supplementary figures and images for: The causal relationship between 41 inflammatory cytokines and hypothyroidism: bidirectional two-sample Mendelian randomization study
Source: Front Endocrinol (Lausanne). 2024 Jan 22;14:1332383. doi: 10.3389/fendo.2023.1332383 (PMC10840409; doi:10.3389/fendo.2023.1332383)

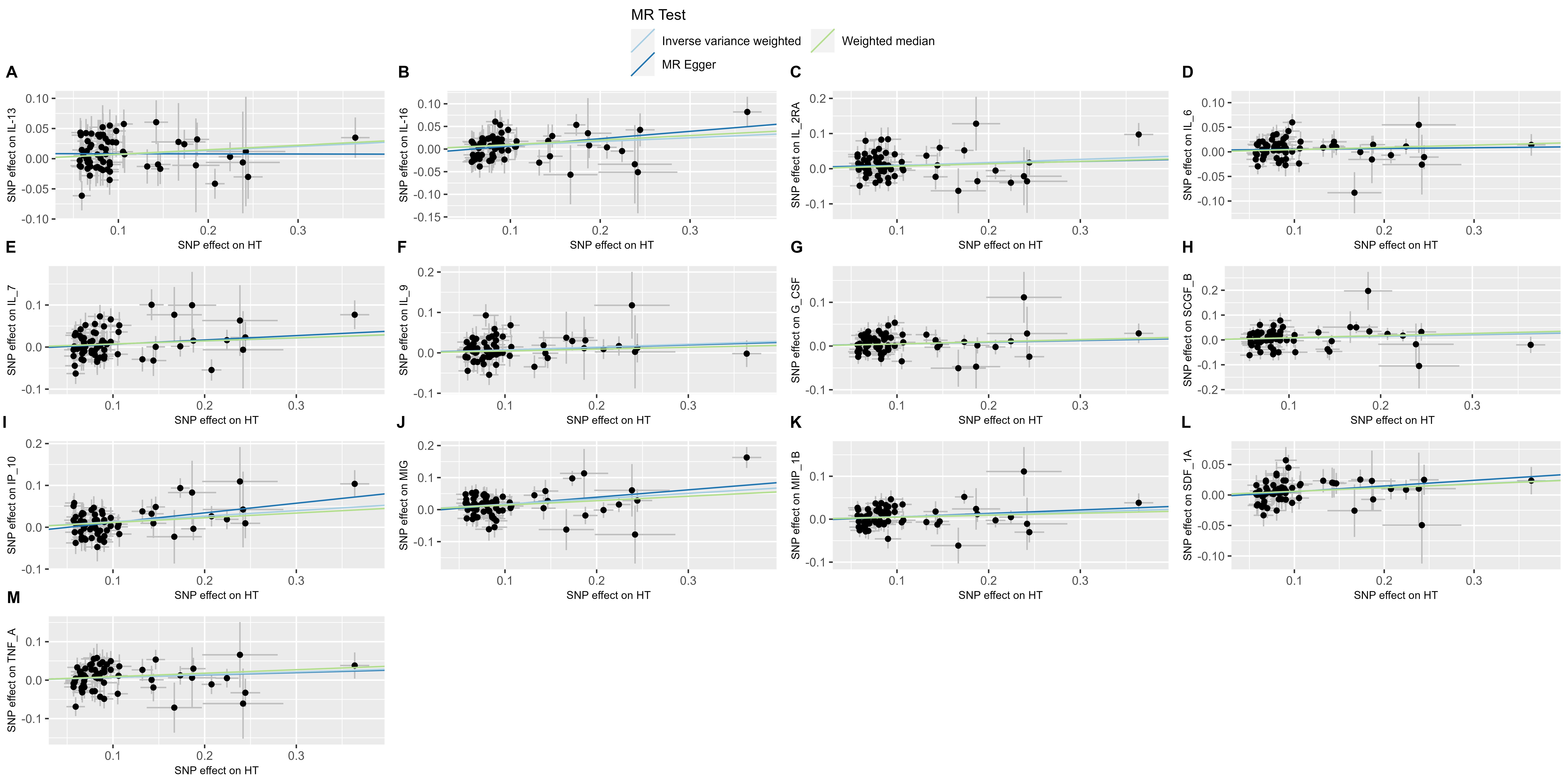

Supplement: Supplementary file 2 [file Image_4.jpg]

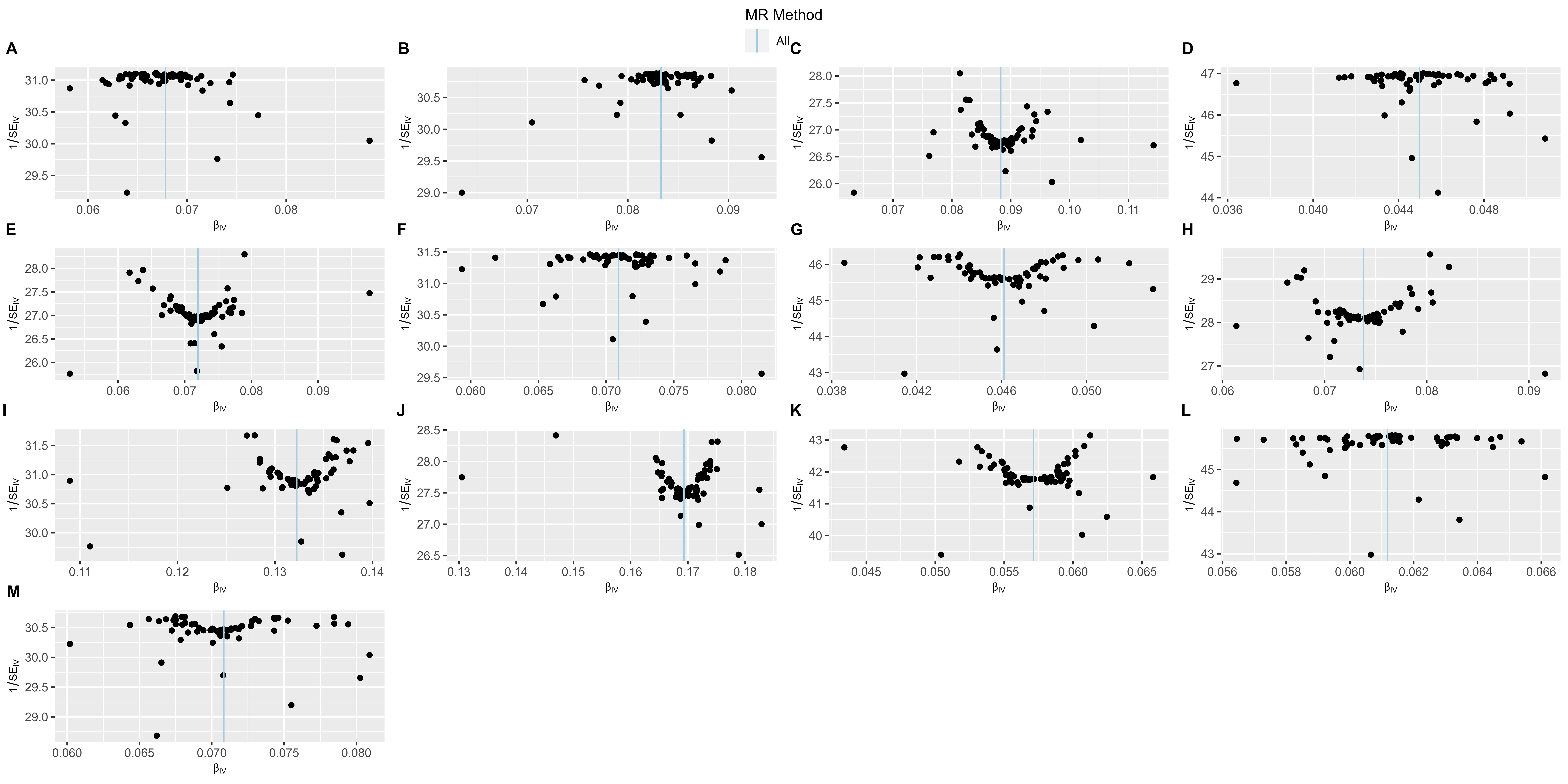

Supplement: Supplementary file 3 [file Image_5.jpg]

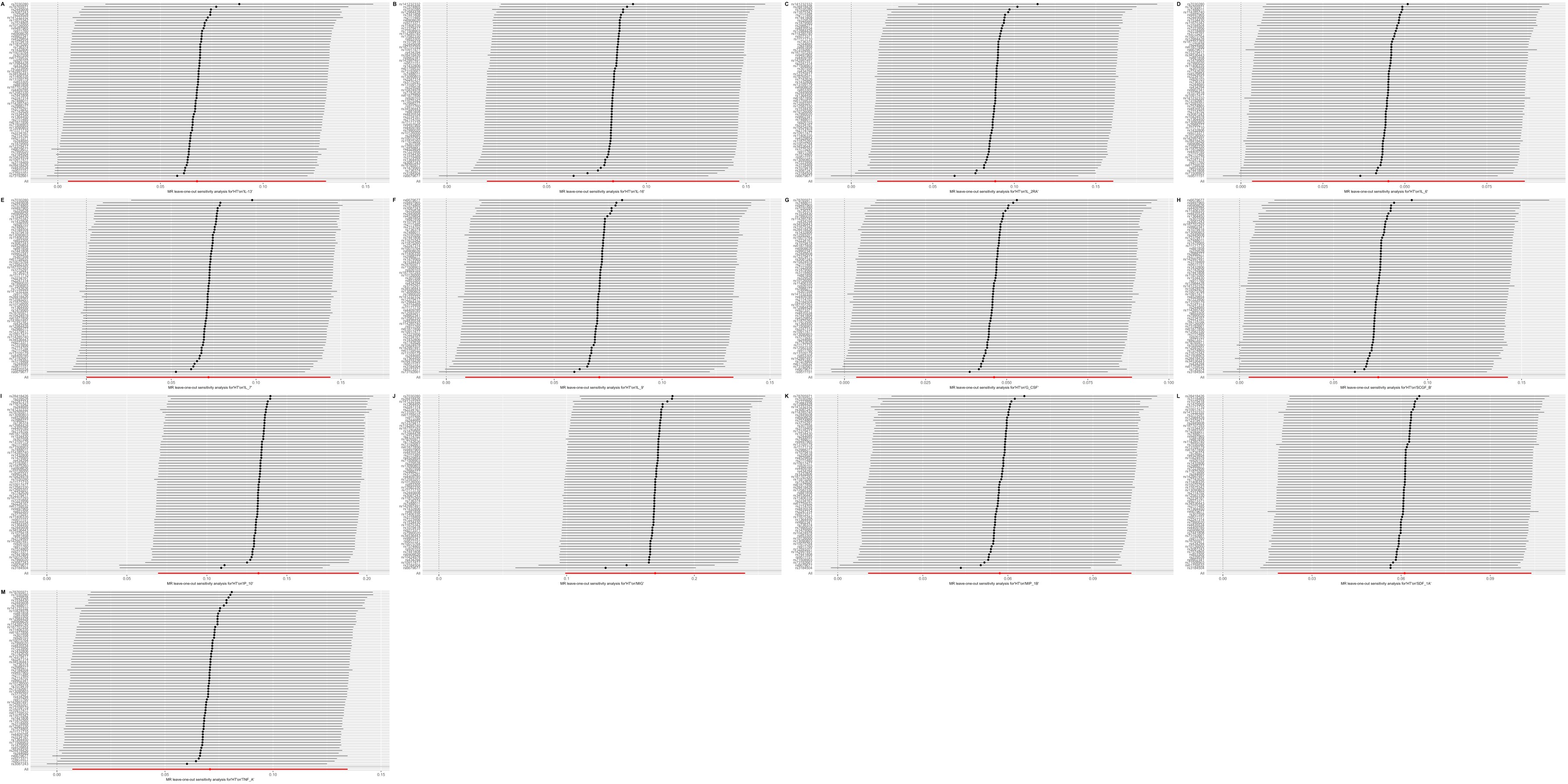

Supplement: Supplementary file 4 [file Image_6.jpg]
